# Different views of medical and nursing professionals on the methods for glycaemic control implemented in intensive care. a cross-sectional study in seven hospitals within the uk

**DOI:** 10.1186/2197-425X-3-S1-A927

**Published:** 2015-10-01

**Authors:** R Fernandez-Mendez, R Windle, GG Adams

**Affiliations:** University of Nottingham, School of Health Sciences, Nottingham, United Kingdom

## Introduction

Stress hyperglycaemia in critical patients can be fatal, but its management in intensive care units (ICUs) has not yet been standardised [1]. Critical care professionals are key stakeholders in research for the quality improvement of clinical practice within the ICU [2]. The GlyCon study is a multiple methods study which includes a survey to professionals, conducted in seven ICUs within a UK-based ICU network.

## Objectives

The purpose of the survey was to describe the opinions of intensive care nursing and medical staff about their methods for glycaemic control, and to explore possible associations between these opinions and the professionals' roles and level of experience in intensive care.

## Methods

An online survey was sent to all nursing and medical staff of the seven ICUs. The survey included questions on effective glycaemic control, treatment of varying degrees of hypoglycaemia, and deviations from protocols' recommendations.

## Results

Forty professionals answered the survey. Regardless of their role or level of experience, most professionals (77.5%) stated that they would treat a hypoglycaemia of 2.2 - 4 mmol/L with glucose only depending on the patient's underlying condition. After controlling for their level of experience, medical doctors were over 20 times more likely to rate “a patient spending less than 50% of the admission time within the glycaemic target range” as “poor glycaemic control” than their nursing colleagues (Adjusted odds ratio, Adj OR>20, p < 0.011). On the other hand, after controlling for their level of experience, nurses were more likely to rate pre-specified deviations from protocols' recommendations as “major” than their medical colleagues. Among others, nurses were around 17 and 11 times more likely to rate “an insulin infusion restarted 2 or less hours late” (Adj OR = 17.1, p = 0.005), and “giving an incorrect amount of rescue glucose once” (Adj OR = 10.9, p = 0.036), respectively, as “major devia tions” as compared with their medical colleagues.

## Conclusions

Professionals' views on various aspects of glycaemic control in intensive care were associated with their profession and level of experience. This study suggests that these factors must be accounted for in research studies looking at the effectiveness and safety of methods for glycaemic control. A larger study is necessary to confirm the results of this pilot survey.

## Grant Acknowledgment

GlyCon study is a PhD study funded by Nottingham University Hospitals Trust, and has also received the support of the School of Health Sciences of the University of Nottingham.

**Figure 1:**
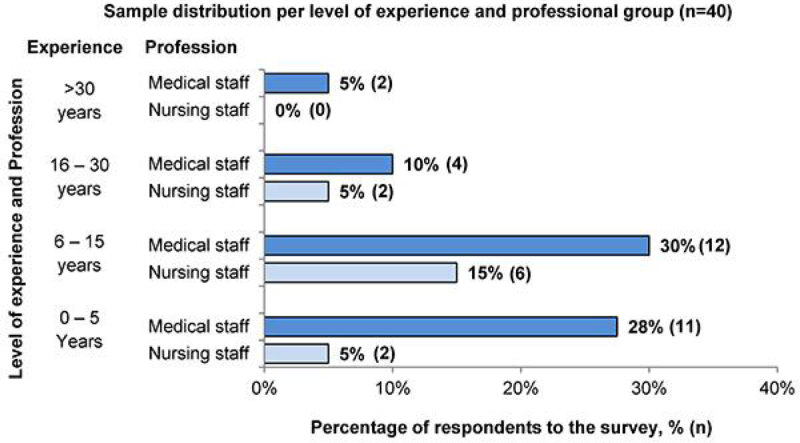
Figure 1

**Figure 2:**
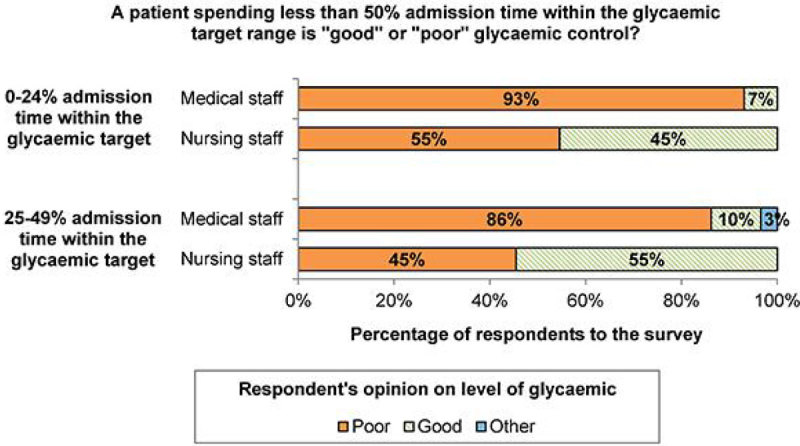
Figure 2
